# Intact glycosphingolipidomic analysis of the cell membrane during differentiation yields extensive glycan and lipid changes

**DOI:** 10.1038/s41598-018-29324-7

**Published:** 2018-07-20

**Authors:** Maurice Wong, Gege Xu, Dayoung Park, Mariana Barboza, Carlito B. Lebrilla

**Affiliations:** Department of Chemistry, University of California, Davis, 1 Shields Ave., Davis, California 95616 USA

**Keywords:** Lipidomics, Mass spectrometry, Bioanalytical chemistry, Glycomics

## Abstract

Glycosphingolipids (GSLs) are found in cellular membranes of most organisms and play important roles in cell-cell recognition, signaling, growth, and adhesion, among others. A method based on nanoflow high performance liquid chromatography-chip-quadrupole-time-of-flight mass spectrometry (nanoHPLC Chip-Q-TOF MS) was applied towards identifying and quantifying intact GSLs from a variety of samples, including cultured cell lines and animal tissue. The method provides the composition and sequence of the glycan, as well as variations in the ceramide portion of the GSL. It was used to profile the changes in the glycolipidome of Caco-2 cells as they undergo differentiation. A total of 226 unique GSLs were found among Caco-2 samples from five differentiation time-points. The method provided a comprehensive glycolipidomic profile of a cell during differentiation to yield the dynamic variation of intact GSL structures.

## Introduction

Glycosphingolipids (GSLs) are a group of complex lipids that are comprised of a ceramide (N-acylsphingosine) glycosidically linked to a glycan moiety. These molecules are ubiquitous in cell membranes, where they are known to participate in cellular processes such as signaling, adhesion, and cell differentiation, among other functions^1^. Malignancies such as cancer and lysosomal storage diseases are also associated with marked changes in the glycolipidome of affected cells. Tumor cells express aberrant glycosylation in GSLs, displaying either incomplete synthesis leading to an accumulation of precursors, or further addition of glycan residues to form new structures^2^. Furthermore, some cancers have been observed to shed elevated levels of GSLs with immunosuppressive activity, and the rate of shedding is affected by the length of the acyl chain in the ceramide^3,4^. Thus there is value in determining the levels of intact GSLs and identifying both its glycan and lipid portions.

Caco-2, a human colorectal cancer cell line, undergoes enterocyte-like cellular differentiation *in vitro*. As such, Caco-2 has been used to model drug absorption in the human intestine and to study interactions between gut microbiota and intestinal epithelial cells^5,6^. Previous studies have shown that intestinal epithelial cells have a varied array of glycolipids, including neutral, sulfated, sialylated, and fucosylated GSLs, as well as globo-type GSLs^7,8^. Furthermore, these GSLs also express ABO blood group and Lewis epitopes^9^. Clinical studies on colorectal cancer tissue have found alterations in GSL glycosylation during cancer progression, such as increased fucosylation and a shift from type I (lacto-type) to type II (neolacto-type) oligosaccharides^10,11^. Studies on the N-linked glycosylation of Caco-2 have revealed that undifferentiated cells express abundant high-mannose structures while differentiated cells have mostly complex and hybrid structures decorated with fucose and sialic acid^12^. The expression of blood group antigens on the glycoproteins of differentiating Caco-2 has also been explored^13^. However, we do not yet have a clear picture of how the cell surface GSLs of Caco-2 change in relation to differentiation, which may yield insights into the link between cell surface glycosylation and cancer progression.

Despite its potential for drug and biomarker discovery, a comprehensive analysis of the cell surface glycosphingolipidome can often prove challenging due to the structural complexity and heterogeneity of GSL structures, as well as the amphipathic nature of these biomolecules. The first challenge arises from the extraction and purification of GSLs from biological matrices. The simplest and most common approach is through solvent extraction; the procedures described by Folch^14^ and Bligh and Dyer^15^ are now well-established and widely used. In both procedures, total lipids are first extracted from the sample, and then partitioned between a biphasic solvent system. Charged groups or those with at least four glycan residues mostly partition to the methanol-rich layer, while other less polar lipids remain in the chloroform-rich layer^16^. When collecting the methanol-rich layer, recoveries greater than 90% can be achieved for sialylated GSLs, as reported by Svennerholm^17^. However, neutral GSLs with less than four glycan residues are not effectively recovered^16^. In this report, we utilize a modified Folch procedure and focus mainly on the GSLs that are recoverable from the methanol-rich layer. Although the limited recovery of mono-, di-, and tri-hexosylceramides will cause their abundances to be underreported, we nevertheless include them in our analysis to follow the changes in their abundance relative to the other samples.

The formation of the glycan is a non-templated process, which can lead to glycan structures that vary not just in monosaccharide composition, but also in sequence, linkage, and branching^18^. The ceramide moiety can have between two to four hydroxyl groups and varying unsaturation and length in their fatty acyl chain^18^. Hence the second challenge is in addressing the heterogeneity of these complicated biomolecules. Early approaches made use of thin-layer chromatography (TLC) for purification and separation, and immunoassay and lectin-binding techniques for quantitative and qualitative analysis of specific glycan motifs^19,20^. However, these approaches are applicable to only a few glycan epitopes for which there are known lectins, and they are unable to reveal the nature of the ceramide. More recent efforts make use of enzymatic digestion to cleave the lipid portion from the glycan headgroups before analyzing the portions separately, at the expense of information about how these two portions are connected^21,22^.

Mass spectrometry, especially when coupled with high performance liquid chromatography, has emerged as the method of choice for characterizing large numbers of GSLs^23,24^. There have been several examples of MS analysis of intact GSLs. Neutral GSLs from human erythrocytes and sialylated GSLs from granulocytes have been analyzed through nano-HPLC/ESI-Q-TOF by Kirsch *et al*.^25,26^. Gangliosides from murine cell lines and human brain have also been examined extensively with the technique^27,28^. Intact gangliosides from milk have been characterized with MALDI-FTICR MS and UHPLC-QqQ MS^29,30^. MALDI-MS has also been performed on intact murine brain gangliosides and permethylated GSLs from differentiated human embryonic stem cells^31,32^. MS profiling of intact gangliosides was performed by Zamfir *et al*. to identify and elucidate ganglioside structures from human astrocytoma, its surrounding tissue, and normal brain tissue using an Orbitrap MS resulting in approximately 80 unique compositions^33^. A study of gangliosides from human serum using nano-HPLC-ESI Q-TOF MS also by Kirsch *et al*. profiled 33 gangliosides and yielded potential biomarkers for pancreatic cancer, illustrating the potential of profiling methods for biomarker discovery^34^.

In this report, we employ a nanoflow high performance liquid chromatography chip-quadrupole-time-of-flight mass spectrometry (nanoHPLC Chip-Q-TOF MS) method to comprehensively identify and quantitate over 220 intact cell-surface GSLs of Caco-2 during differentiation. The method effectively covered 15 different glycan headgroups, including acidic GSLs with sulfation and sialylation, as well as fucosylated and non-fucosylated neutral GSLs. The glycans of each identified GSL were characterized by their composition and connectivity, while the ceramides were distinguished by their lipid chain length, number of hydroxyl groups, and degrees of unsaturation. Furthermore, we applied observed trends in GSL elution towards predicting the retention times of GSLs and assigning peaks with more confidence thereby increasing the coverage significantly. The study presents the most comprehensive analysis of cell membrane GSLs to date, and exceeds the number of identified GSLs in earlier reports by a factor of 2. It further yields highly reproducible and quantitative information allowing measurement of distinct changes during cellular progression.

## Results and Discussion

Caco-2 cells undergo enterocyte-like differentiation upon reaching confluence. Although the cell line comes from colorectal cancer, differentiated Caco-2 cells acquire morphological and functional features that resemble intestinal epithelial cells. During differentiation, they exhibit changes such as bicellular tight junction assembly and remodel the apical surface to form a brush border^5^. As the cells reach confluence, they stop dividing exponentially and begin to differentiate, with cells forming villi and migrating to the tip as they mature^35^. The cell surface glycosylation of proteins was previously reported to change during the differentiation process, based on the analysis of released N-glycans^12^.

In this study, cell surface GSLs were monitored *in vitro* at five different time points: undifferentiated (Day 5), confluent (Day 7), partially differentiated (Day 14), differentiated (Day 21), and post-differentiated (Day 24). Analysis of MS data yielded a total of 227 unique GSLs in all the Caco-2 samples as characterized by a combination of accurate masses, retention times, and tandem MS. Peak areas of identified GSLs are normalized to the total abundance of GSLs in each sample and reported as relative abundances.

### LC-MS/MS data analysis

**Figure 1:**
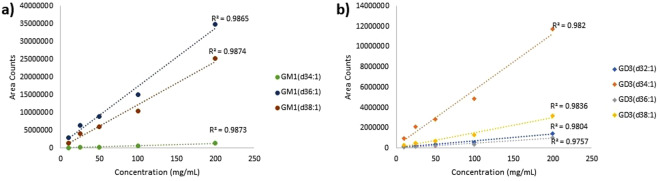
Linear regression plots for the area counts of GSL compounds from (**a**) GM1a and (**b**) GD3 standards as a function of concentration. The peak area shows generally linear behavior with concentration. The coefficient of determination for each identified GSL is shown.

Ganglioside standards GM1a and GD3 from Calbiochem and Avanti respectively were run with the samples to provide the retention times standards. The commercial compounds were found to contain mixtures of different ceramide chain lengths, and their retention times matched those identified from samples. The GM1a and GD3 standards were also run repeatedly during the batch analysis to determine instrument reproducibility over time. The relative standard deviations of GM1a and GD3 were calculated to be 19.2% and 16.5% respectively from 5 injections over 24 h of instrument run time. Serial dilutions of the standards from 10ug/ml to 200 ug/ml were also injected to determine the linear range of the instrument response. Figure 1 shows the linear plots of the peak area for the GSLs identified from the standards as a function of concentration. The linear range encompasses the response of the reported GSLs, assuming that the response of the different GSLs are comparable.

**Figure 2:**
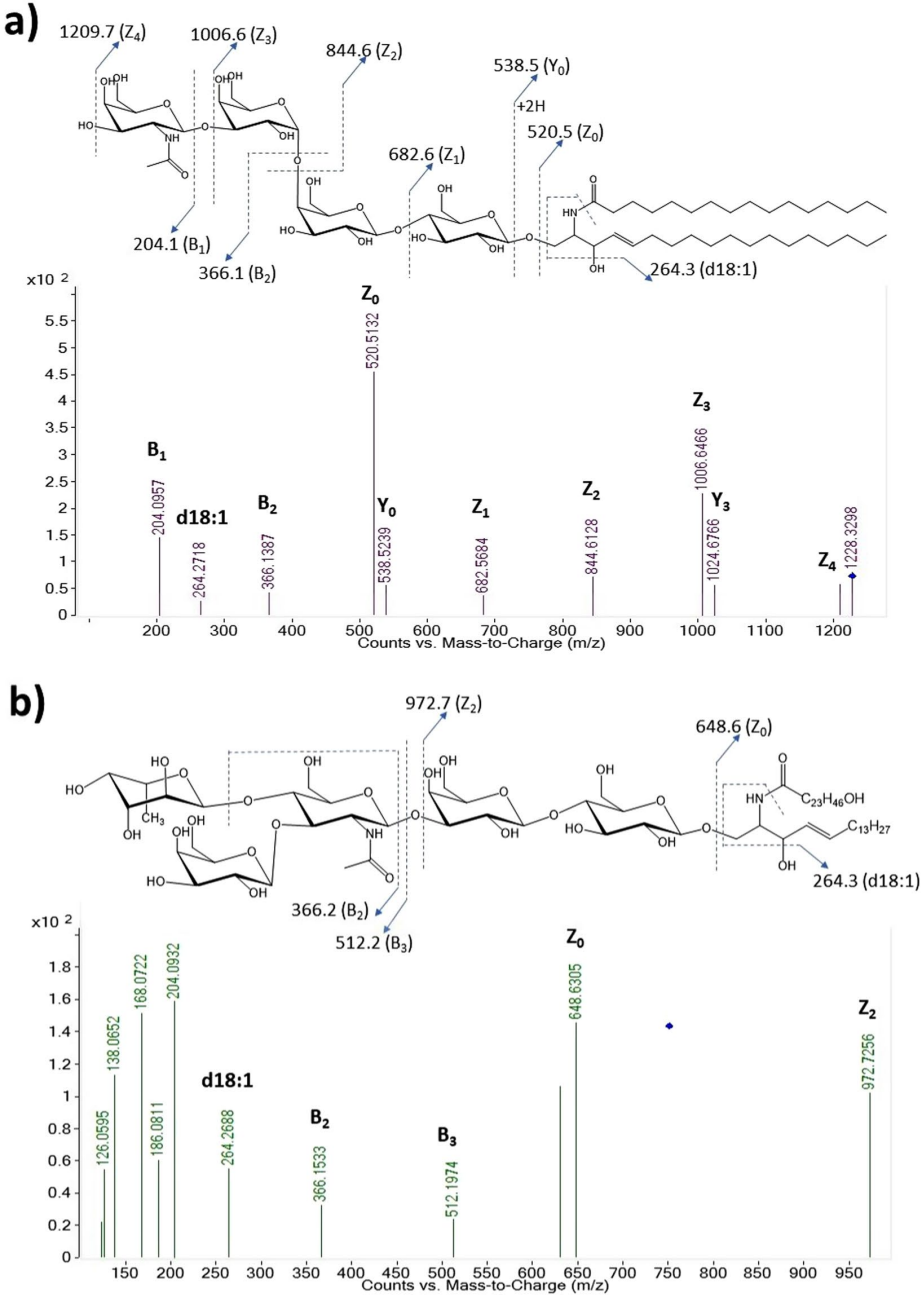
Tandem MS spectra of intact GSLs (**a**) Gb_4_(d18:1/C16:0), and (**b**) Fuc-Lc_4_(d18:1/C24:0 OH) with the fragmentation pattern annotated for relevant peaks. Putative structures of the compound are also presented with designations for product ions.

GSLs were initially identified based on the monoisotopic masses of possible combinations of glycans and ceramides. However, identification of GSL structures from cells by MS data can be complicated by the presence of phospholipids and other lipid species, which cannot be completely separated through Folch extraction. These compounds can have masses that differ by only a few ppm with those of hexosylceramides, lactosylceramides, and sulfatides. To avoid the misidentification of GSLs, their structures were verified with tandem MS data using collisional-induced dissociation (CID). As was shown previously by others, ions corresponding to combinations of ceramide fragments and glycan residues such as hexose, fucose, N-acetylhexosamine, and N-acetylneuraminic acid were featured in the CID spectra^36^. Ceramides, which are composed of a sphingoid base and a fatty acid, were found to have mostly 2 to 3 hydroxyl groups, 32 to 44 total carbon atoms, and 0 to 2 hydrocarbon unsaturation based on this analysis. Sphingosine, an 18-carbon aliphatic amine with an unsaturated hydrocarbon chain and 2 hydroxyl groups, is the most common sphingoid base found in mammals, although variations in chain length, unsaturation, and the number of hydroxyl groups are also present^18^. Fragment ions for the sphingoid moieties *d18:1*, *d18:0*, and *d20:1* were found at *m/z* 264.3, 266.3, and 292.3 respectively. Annotated tandem mass spectra of two GSLs in Caco-2 are shown in Fig. 2. The fragmentation pattern in Fig. 2a suggests a tetraosylceramide that has a globo-type structure, Gb_4_(d18:1/C16:0). Compound **2b** has the glycan composition Hex_3_HexNAc_1_Fuc_1_, which can possibly have the Lewis a/x or the H antigen (Lewis x structure is shown). It should be noted that the linkages and absolute configuration of the glycan residues cannot be determined from the mass spectrum alone, and that the proposed structures presented here are putative and based on current knowledge of human intestinal cell surface GSLs^7^.

**Figure 3:**
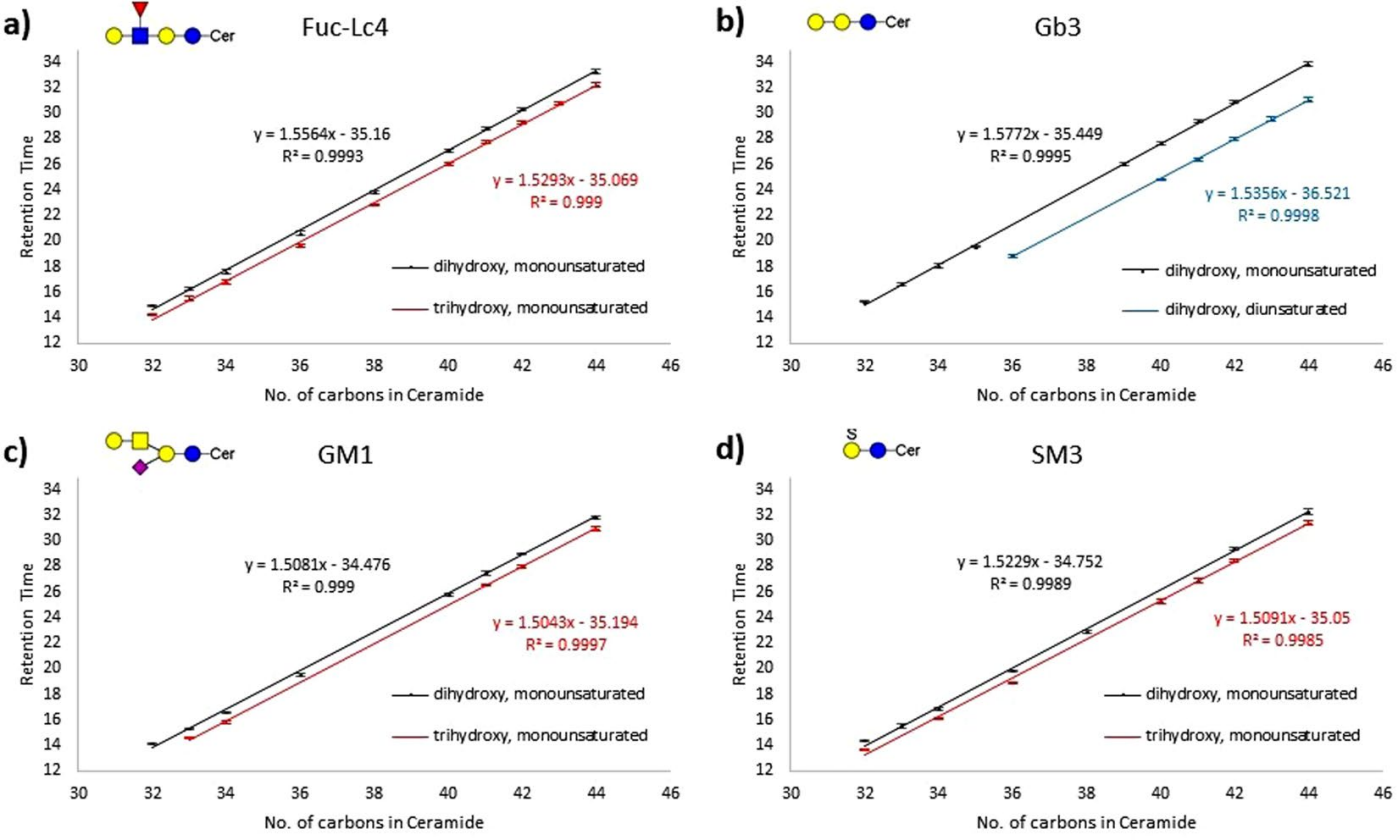
Graphs of retention time (mins) vs the number of carbons in the ceramide for 4 different glycan headgroups. Putative structures are shown for each glycan headgroup. The linear plots of di- and tri- hydroxy monounsaturated ceramides are compared in fuc-Lc_4_ (**a**), GM1 (**c**), and SM3 (**d**), while mono- and di- unsaturated dihydroxy ceramides are compared in Gb_3_ (**b**). These relationship allowed the identification of low abundance GSL with accurate masses, but where tandem MS could not be obtained. This predictive method was used to increase the number of identified compounds.

With our data-dependent acquisition program, we obtained tandem MS spectra for 54% of the reported GSLs. To identify GSLs with greater confidence, especially those that were too low in abundance to obtain tandem MS, we predicted the retention times of GSLs based on observed elution trends of GSLs identified from tandem MS data. These trends were also observed in GM1a and GD3 ganglioside standards which contained a mixture of different lipid chain lengths. The prediction considers the characteristics of both the glycan and the lipid. For GSLs with the same glycan compositions that differ only by the lengths of their hydrocarbon chains in the ceramide, a linear trend was found between the retention times and the number of carbons in the ceramide, as shown in Fig. 3. Lengthening the ceramide by 1 carbon delayed elution by about 1.6 minutes. Those with trihydroxy ceramides eluted 0.6–1 min earlier than comparable dihydroxy ceramides. GSLs with a charged group, such as a carboxylic acid or sulfate moiety, eluted about 1–1.5 mins earlier than a comparable GSL with the same ceramide and the same number of monosaccharide residues.

**Figure 4:**
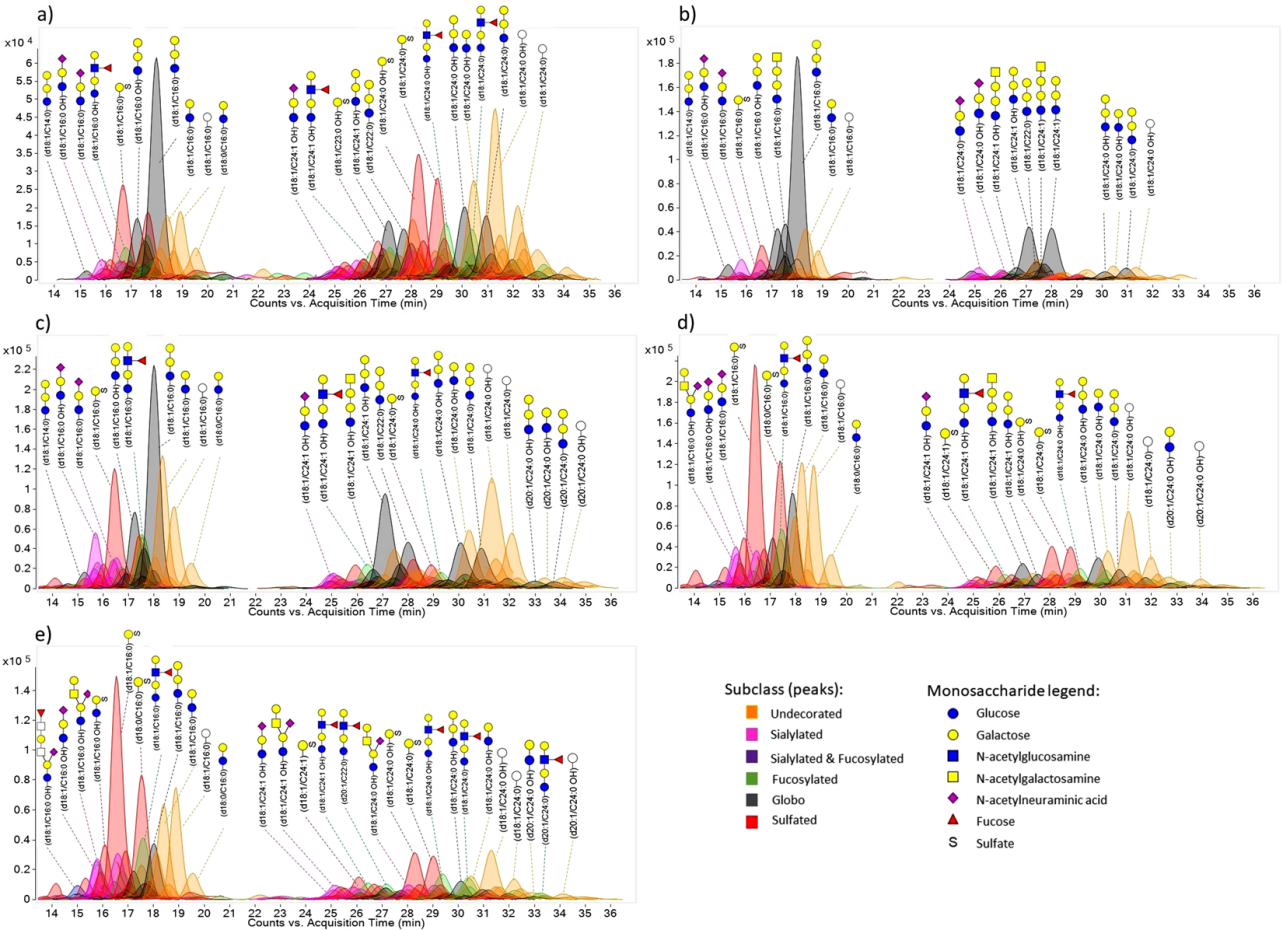
Representative chromatograms of GSLs in (**a**) undifferentiated (Day 5), (**b**) confluent (Day 7), (**c**) partially differentiated (Day 14), (**d**) differentiated (Day 21), and (**e**) post-differentiated (Day 24) Caco-2 cells. A different peak color is assigned to each glycan subtype: Sialylated – pink, Fucosylated – green, Fucosylated & Sialylated – blue, Globoside – gray, Sulfated – red, Undecorated – orange. Peaks of selected GSLs are annotated with schematic representations of their glycan structures while the ceramide structures are indicated above the glycan. The y-axis of each graph is adjusted to the most abundant compound in each time point. Structures were drawn with GlycoWorkbench^42^.

A library of GSLs that includes their monoisotopic masses, chemical formulae, and estimated retention times was created for rapid identification and semi-quantitation. The library combines identified glycan head groups with other possible configurations of ceramide to have a more comprehensive list of GSLs. Signals that correspond to the GSLs in the library were extracted by the MassHunter Qualitative Analysis software through its targeted Find by Formula function, and quantitated by their peak area. Peaks were extracted and quantitated with a mass tolerance of 10 ppm and a RT tolerance of 0.5 min. The program also accounts for the isotopic distribution of the compounds, their charge states, and the presence of other adducts for peak quantitation. Charge states of 1–2 were included in peak extraction, and both proton and ammonium adducts were considered as well. For data analysis, abundances are normalized to the total abundance of identified GSLs in each sample. Relative abundances are reported for each GSL. Representative extracted compound chromatograms for each of the five differentiation time points are shown in Fig. 4, annotated with schematic representations of the more abundant GSLs. A complete list of the GSL compositions, retention times, and abundances are provided in Supplementary Table 1.

### Glycan profile changes

**Figure 5:**
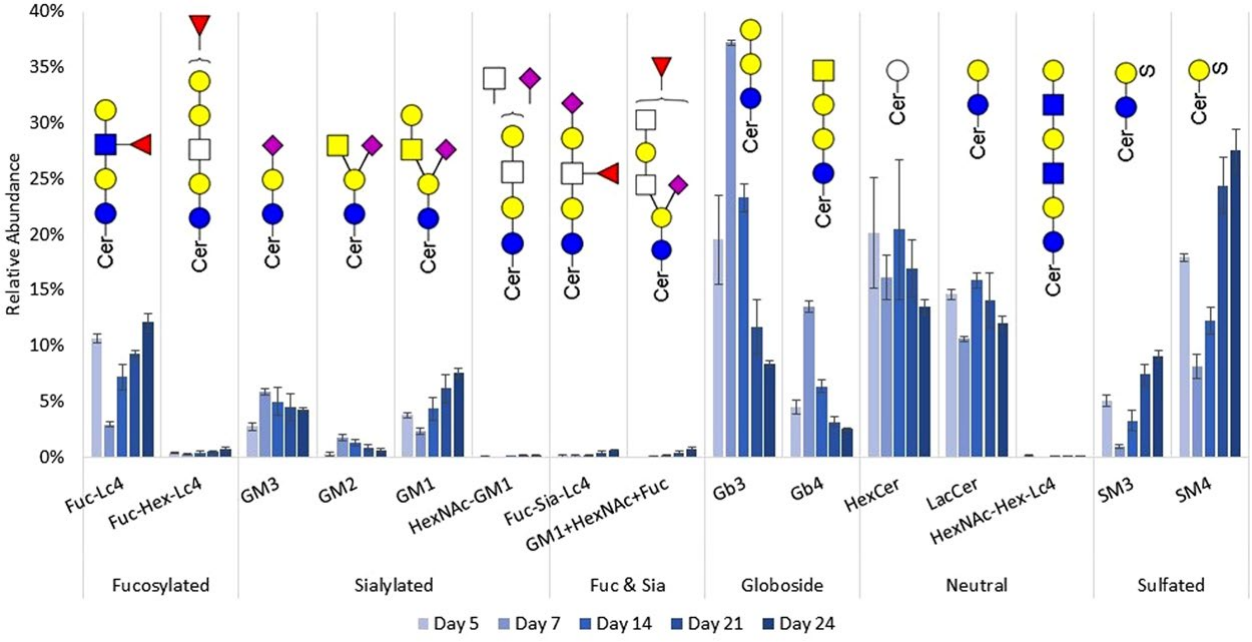
Relative abundances of GSL glycans. Error bars indicate the standard deviation for 3 biological replicates at each differentiation time point.

The lipid-bound glycan profile is not only tissue-specific, but is also affected by the condition and development of the cell^18^. There is a surprisingly small number of glycan compositions that comprise the glycolipidome. A total of 15 glycan compositions were identified and quantitated. A summary of the glycan structures and their abundances relative to the total abundance of all GSLs found in each sample is shown in Fig. 5. GSLs with isomeric glycan units, such as linkage and epimeric isomers, do not resolve sufficiently on the C18 column, and some representations will likely correspond to several isomeric structures. The putative structures of the glycans shown here are believed to be the major contributors based on previous studies on the GSLs in human intestinal tissue^7,10^.

**Figure 6:**
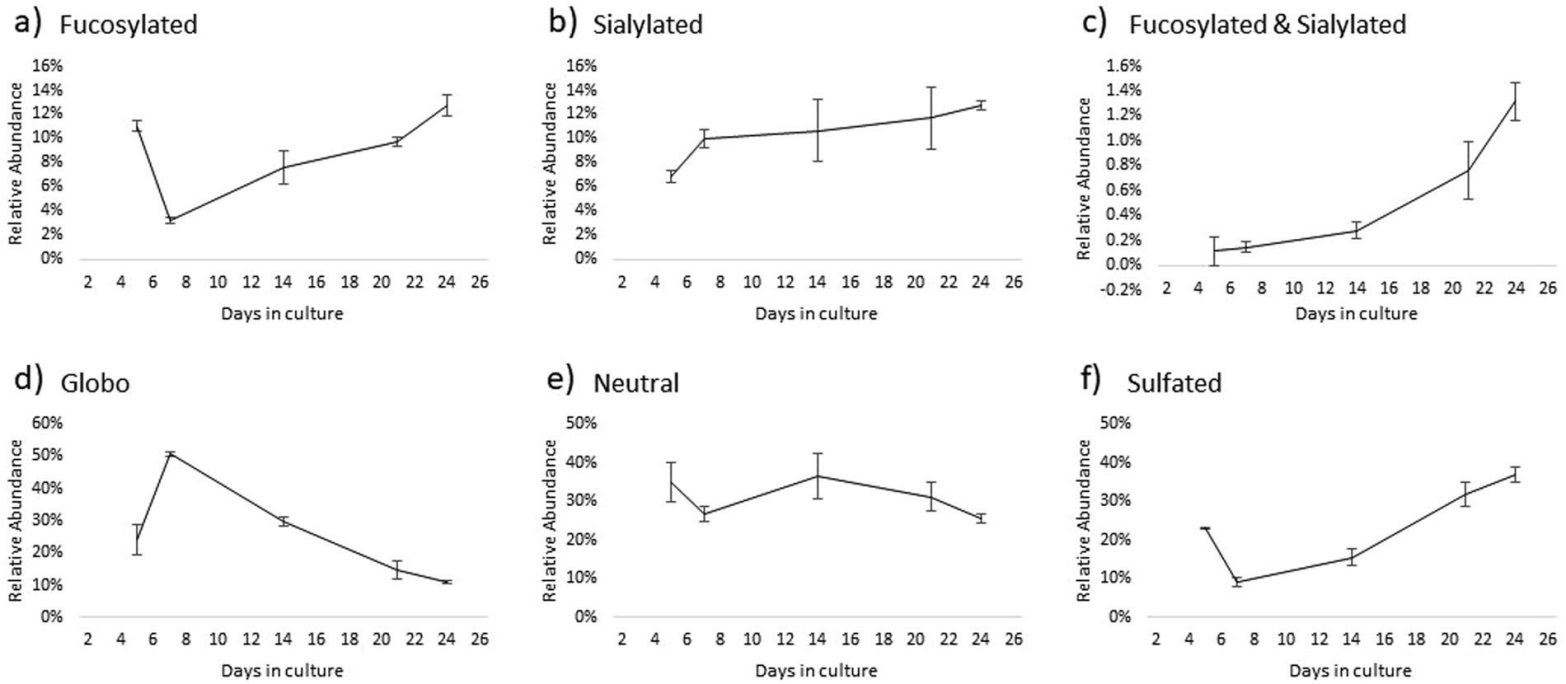
Relative abundances of GSLs based on glycan subtype as a function of growth time. Error bars indicate the standard deviation for 3 biological replicates.

GSLs were further grouped according to glycan subtypes to have a more general view of how the cell surface GSLs change during differentiation. The general trends for each subtype are shown in Fig. 6. The subtypes presented here are mostly based on how glycans are decorated with specific monosaccharide moieties, such as sialic acid and fucose, which are frequent features in glycan-based antigen epitopes.

Fucosylated structures initially comprised 11% of GSLs in undifferentiated cells. This number fell to 3.2% during confluence, then climbed back up to 12.8% in post-differentiated cells. These structures may express Lewis a/x epitopes, both of which have been found in colon cancer^37^. Sialylated GSLs steadily increased throughout the differentiation timeline. However, the gangliosides GM3 and GM2 followed a decreasing trend while GM1 increased from confluence until differentiation. GM3 at confluence accounted for 5.9% of total detected GSLs and tapered off to 4.3% post-differentiation, while GM1 increased from 2.3% to 7.6% over the same period. Caco-2 has been reported to express mostly GM3 on its cell surface. The expression of GM1 may be due to a passage-dependent cell transformation that promotes the epithelial to mesenchymal transition (EMT) of the cultured cells^38^. This concomitant but undesirable transformation can further complicate reproducibility studies between passages as it can drastically change the lipid-bound glycosylation pattern. GSLs that were decorated with both fucose and sialic acid also showed a steady increase from 0.12% (undifferentiated) to 1.32% (post-differentiated). The fragmentation spectra suggest that the GSLs with Hex_3_HexNAc_1_Fuc_1_NeuAc_1_ (labeled Fuc-Sia-Lc_4_ in Fig. 5) mostly express sialyl-Lewis a/x epitopes.

Globosides were observed to predominate as the Caco-2 cells reached confluence, accounting for more than 50% relative abundance on day 7 then decreasing to 11% in post-differentiated cells. Both Gb_3_ and Gb_4_ followed the same trend, being at their most abundant during confluence. The marked increase in globosides at confluence depresses the relative abundance of other decorated subtypes, as shown in Fig. 6, after which there is a steady increase in GSLs decorated with fucose or sialic acid. The trend is analogous to the switching of GSL core structures in human embryonic stem cells from globo-type to ganglio-type GSLs upon differentiation^31^. The globoside Gb_3_, which is among the most abundant glycans found in the cells, has been associated with increased metastasis in colon cancer^39^.

Sulfatides are sulfated GSL structures with one or two hexose units. Sulfatides play an important role in cell adhesion, and their elevated expression has been linked with metastatic potential of colorectal cancer^40^. Two sulfatides were monitored: monohexosyl sulfatide, SM4, and dihexosyl sulfatide, SM3. Around 77% of total sulfatide had SM4 structure at all time points except at confluence, when SM4 accounted for 89%. Overall, the subtype decreased from 23.0% to 9.1% upon confluence, then climbed steadily to 36.7% post-differentiation.

Neutral GSLs include monohexosylceramides (HexCer), lactosylceramides (LacCer), and elongated polylactosamines. Folch extraction of HexCer (both glucosyl- and galactosyl- ceramides) and LacCer have low recoveries because of their more nonpolar nature^16^. However, here the changes to their relative amounts can be compared across the differentiation timeline because the solvent ratios were consistent throughout this analysis batch. LacCer is the common precursor for more complex GSLs. Both HexCer and LacCer were at their most abundant during partial differentiation, and declined as GSLs became increasingly decorated during differentiation. Polylactosamines were not consistently found in all samples because of their low abundance.

### Ceramide profile changes

**Figure 7:**
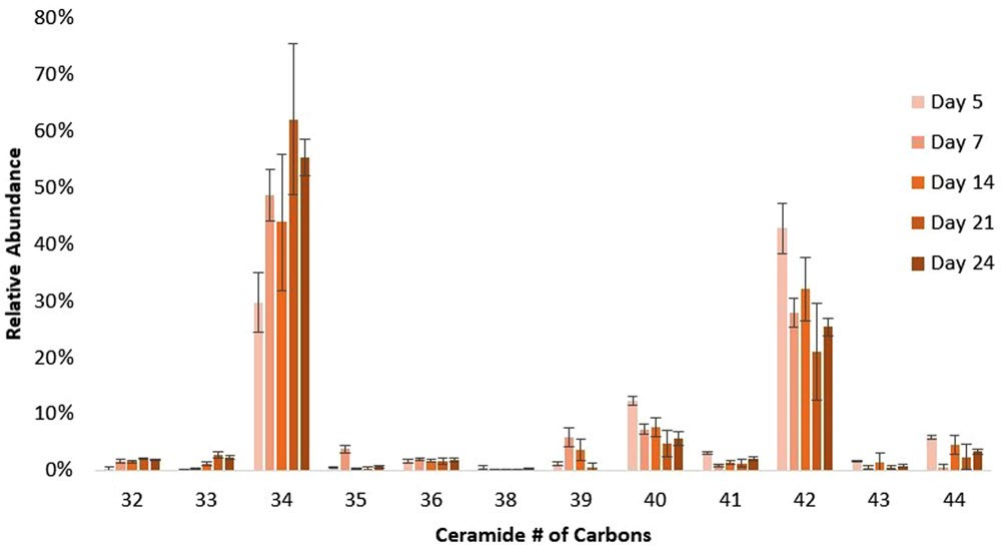
Relative abundances of GSLs according to the total number of carbons in the ceramide. Error bars indicate the standard deviation for 3 biological replicates.

Variations in composition of the ceramide portion, such as the number of hydroxyl groups, the degree of unsaturation, and the chain lengths of the sphingoid and fatty acid, were also observed. The ceramides mostly expressed the d18:1 sphingoid moiety, with low levels of d20:1, and fatty acids with 14 to 24 carbons. Ceramides with 34 and 42 total carbons were the most abundant among the GSLs, as shown in Fig. 7; the ceramides are mostly comprised of the sphingoid d18:1 that are N-acylated with C16:0, C24:0, or C24:1 fatty acids. Ceramides with C16:0 acylation increased from 30% in undifferentiated cells to around 60% in fully differentiated cells, while those with C24:0 or C24:1 decreased from 43% to 25% over the same period. Ceramides with an odd number of carbons were also observed at low abundances. Trihydroxyceramides were also abundant, in which the fatty acid has an additional α-hydroxyl group aside from the two found in sphingosine^18^. No clear trends were found between the relative abundances of dihydroxy- and trihydroxy-ceramides. On average, dihydroxyceramides accounted for 55.9% (±4.7) of all GSLs. Low levels (<1%) of tetrahydroxyceramides were also found in some samples.

**Figure 8:**
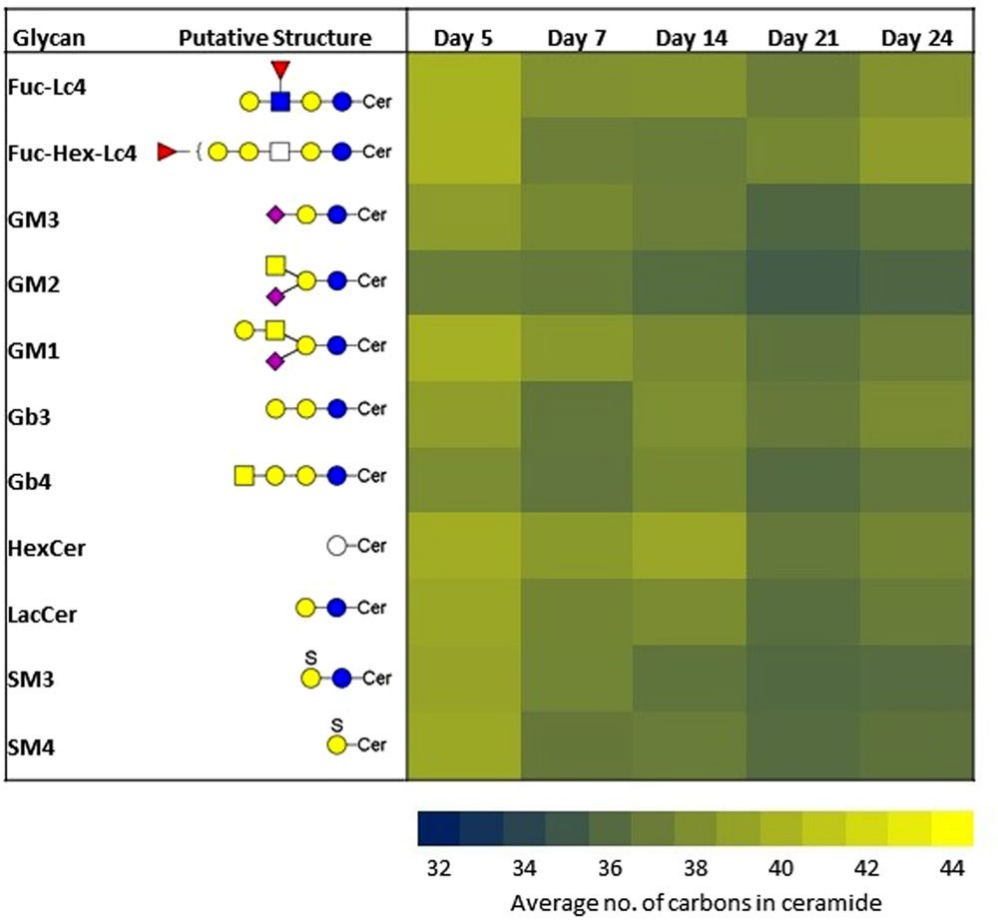
Heat map of the average length of ceramide lipids associated with different glycan headgroups during differentiation.

The changes to the attached lipid for each glycan head group can be tracked as well. The length of the acyl chain is correlated with the rate of shedding and GSL turnover, with shorter acyl chains exhibiting more rapid turnovers than longer ones^41^. Figure 8 shows a heatmap of the average number of carbons in ceramide associated with the different glycan headgroups. In general, the ceramides became shorter until full differentiation on Day 21, then lengthened again as they approached post-differentiation. This suggests that GSL turnover was fastest when the cells are fully differentiated.

## Materials and Methods

### Cell culture

Caco-2 cells were obtained from American Type Culture Collection and cultured in Eagle’s Minimum Essential Medium, supplemented with 10% (v/v) fetal bovine serum, 100 U/ml penicillin, and 100 ug/ml streptomycin. The medium was replaced every 2–3 days, and cells were subcultured on reaching around 80% confluence using 0.05% trypsin (Gibco). Cells were grown at 37 °C in a humidified incubator with 5% CO_2_. Cells were collected in biological triplicates by scraping after 5, 7, 14, 21, and 24 days, corresponding to undifferentiated, confluent, partially differentiated, differentiated, and post-differentiated cells respectively. All cells were harvested at passage 5. Cell counts of at least 3 × 10^5^ were used for each replicate.

### Membrane extraction

Harvested cells were suspended in homogenization buffer containing 1:100 protease inhibitor cocktail set V, 0.25 M sucrose, and 20 mM HEPES, with the pH adjusted to 7.5 using KOH. The cells were lysed with a probe sonicator. Nuclear fractions were pelleted and discarded through centrifugation at 2,000 × *g* for 10 min. Membrane fractions were then pelleted and separated from other subcellular components through a series of ultracentrifugation steps at 200,000 × *g* for 45 min.

### Folch extraction

Pelleted membrane fractions were resuspended in 150 ul water. Methanol and chloroform were then added stepwise to form a mixture of 3:8:4 (v/v/v) water/methanol/chloroform. Samples were homogenized with sonication for 10 min, and centrifuged at 20,000 × g for 1 min. Pellets, which contain mostly precipitated proteins, can be set aside for other analytical procedures, while supernatant portions were collected for GSL analysis. To the supernatant, 100 ul of 0.1 M potassium chloride was added to cause phase separation between the water- and methanol-rich upper layer, which contains mostly GSLs, and the chloroform-rich lower layer, which contains mostly other lipids such as cholesterol and phospholipids. The upper Folch layer was collected and dried under vacuum.

### Solid phase extraction

Samples were further purified using C8 solid phase extraction (SPE) plate. SPE columns were rinsed with 1:1 methanol/water and 1:1 methanol/isopropanol, then conditioned with 1:1 methanol/water. Samples were loaded and washed with 1:1 methanol/water to remove salts. Elution was done with 1:1 methanol/isopropanol, after which the eluted sample was dried.

### GSL standards

Ganglioside standards GM1a (Cat. no. 345724-1MG, Lot no. D00167174) from bovine brain and GD3 (Cat. No. 860060 P, Lot. No. GD3-10) from bovine milk were obtained from Calbiochem and Avanti Polar Lipids, respectively. Standards were diluted to the desired concentrations with 1:1 methanol/water and injected into the LC-MS instrument without further processing.

### NanoHPLC Chip-Q-TOF MS analysis

Samples were reconstituted in 50 ul solution of 1:1 methanol/water and analyzed with an Agilent 6520 Accurate Mass Q-TOF LC/MS equipped with a C18 microfluidic chip, which incorporates an enrichment column, an analytical column, and a nanoelectrospray tip in a single assembly. A binary gradient consisting of (A) 20 mM ammonium acetate and 0.1% acetic acid in water, and (B) 20 mM ammonium acetate and 0.1% acetic acid in 85:15 (v/v) methanol/isopropanol was used to separate the GSLs at a flow rate of 0.3 ul/min. The acquisition method was programmed to linearly increase the percentage of elution solvent B from 80% to 100% over 36 min. One MS and six tandem MS spectra were acquired every 3.1 s on positive mode through data-dependent acquisition. Fragmentation was achieved by collision-induced dissociation (CID) with nitrogen gas at collision energies of around 25V. Data was processed with Agilent MassHunter B.07 software. GSL structures that were identified through their m/z and MS/MS fragmentation pattern were added to a library, which includes their chemical formulae, retention times (RT), and accurate masses. Compounds which match expected GSL masses, but for which there is no MS/MS data, were included when RT can be inferred from confirmed GSL compounds. Configurational isomers were disambiguated based on expected RT and MS/MS data. Compounds were then extracted using the “Find by Formula” program, allowing for 10 ppm mass tolerance and 0.5 min RT tolerance. Charge states of 1–2 and both proton and ammonium adducts were included in the extraction.

### Accession codes

Caco-2: https://www.atcc.org/Products/All/HTB-37.aspx.

## Electronic supplementary material

Supplementary Table 1
