# Author Correction: Intact glycosphingolipidomic analysis of the cell membrane during differentiation yields extensive glycan and lipid changes

**DOI:** 10.1038/s41598-020-78291-5

**Published:** 2020-12-01

**Authors:** Maurice Wong, Gege Xu, Dayoung Park, Mariana Barboza, Carlito B. Lebrilla

**Affiliations:** Department of Chemistry, University of California, Davis, 1 Shields Ave., Davis, CA 95616 USA

Correction to: *Scientific Reports* 10.1038/s41598-018-29324-7, published online 20 July 2018

This Article contains an error. A Data Availability section was originally not included – it should appear as below:

**Data Availability**

Raw mass spectrometry data is freely available and can be found on the MassIVE repository (https://doi.org/doi:10.25345/C51J1Q).

